# Retrospective Analysis of Incidental Myocardial Perfusion Defects on Non-ECG-Gated Contrast-Enhanced CT in Emergency Settings

**DOI:** 10.3390/medicina62020277

**Published:** 2026-01-28

**Authors:** Jia-Hao Zhou, Meng-Yu Wu, Jong-Kai Hsiao

**Affiliations:** 1 Department of Medical Imaging, Taipei Tzu-Chi Hospital, Buddhist Tzu-Chi Medical Foundation, New Taipei City 220811, Taiwan; charles198217@gmail.com; 2 Department of Medical Imaging and Radiological Sciences, Tzu Chi University, Hualien 970374, Taiwan; 3 Department of Emergency Medicine, Taipei Tzu Chi Hospital, Buddhist Tzu Chi Medical Foundation, New Taipei City 220811, Taiwan; skyshangrila@gmail.com; 4 Department of Emergency Medicine, School of Medicine, Tzu Chi University, Hualien 970374, Taiwan; 5 Department of Radiology, School of Medicine, Tzu Chi University, Hualien 970374, Taiwan

**Keywords:** acute myocardial infarction, computed tomography, non-ECG-gated

## Abstract

*Background and Objectives:* Coronary heart disease is a leading cause of death in developed countries. While ECG-gated coronary CT is commonly used to detect coronary artery stenosis, the potential of non-ECG-gated CT (NECE-CT) to reveal incidental myocardial perfusion defects indicative of acute myocardial infarction (AMI) remains underexplored, particularly in emergency settings where rapid diagnosis is crucial. *Materials and Methods:* We retrospectively analyzed 22 suspected AMI patients from the emergency department who underwent NECE-CT without either an initial AMI diagnosis or available cardiac enzyme or ECG data. *Results:* AMI was confirmed in 45% (*n* = 10) of patients, with 30% (*n* = 3/10) showing elevated troponin I levels only after the CT exam. In the AMI group, all patients had perfusion defects, with 20% (*n* = 2) showing transmural defects and 80% (*n* = 8) showing endocardial defects. In contrast, all patients in the non-AMI group exhibited endocardial defects. Coronary artery calcification was significantly higher in the AMI group (70%) compared to the non-AMI group (25%, *p* < 0.05). *Conclusions:* These findings suggest that NECE-CT may reveal myocardial perfusion defects as an ancillary sign of AMI. While not a standalone diagnostic tool, careful evaluation of the myocardium in emergency CT scans may raise suspicion of AMI in patients with atypical presentations, offering more insight than standard methods. Further prospective studies with larger cohorts are needed to validate the clinical utility of these incidental findings.

## 1. Introduction

Acute myocardial infarction (AMI) is a severe manifestation of cardiovascular disease, with increased incidence and mortality rates in Europe, the USA, and northern Asia [1,2,3,4]. It is the single leading cause of death in Europe, accounting for 19% and 20% of deaths in men and women in 2021 [2]. The important pathophysiologic mechanisms of acute coronary syndrome (ACS) begin with atherosclerosis [5] and progress to ischemia. In the diagnosis of AMI, early electrocardiograms (ECGs) are necessary to detect ST-elevation myocardial infarction (STEMI) or abnormal rhythms. Serial ECG and cardiac enzyme measurement are suggested in high-risk ACS patients: in non-ST-elevation acute coronary syndrome (NSTE-ACS), elevated serum levels of cardiac troponin T (cTnT) and I (cTnI) signal ischemia; if these levels are not elevated, repeat measurements are recommended after three to six hours, as troponin peaks increase after three to six hours of ischemia [6,7].

A recently well-established cardiac enzyme, high sensitive troponin I (hsTnI), has been used extensively in Europe for over a decade, ahead of deployment in the USA and other countries [8,9]. In ischemic conditions, the accumulation of reactive oxygen species leads to myocyte cell death [10], which releases cTnT and cTnI, components of the cardiac contractile apparatus interacting with actin and myosin [11]. As unique biomarkers from myocytes, they are used in a highly specific test for AMI. After myocardial injury, cTnT and I are detectable within two to three hours and peak within twenty-four to twenty-eight hours. Currently, clinical practice for evaluating acute coronary syndrome (ACS) patients relies on cTnT/I and electrocardiographic (ECG) myocardial infarction criteria. According to the 2020 European Society of Cardiology NSTE-ACS guidelines, the high-sensitivity cardiac troponin I (hs-cTnI) test should be considered within one or three hours to rule out AMI [12], meaning patients typically stay in the emergency department for four to six hours, undergoing serial evaluation of cardiac biomarkers and ECGs. Early diagnosis and timely primary percutaneous coronary intervention are suggested to enable clinicians to reverse myocardial infarction and prevent catastrophic outcomes [13].

Coronary computed tomography is an alternative, non-invasive diagnostic test widely used to evaluate stenosis of the coronary arteries that has proven its efficacy in reducing chest-pain-related death, as well as reducing resource utilization [14,15,16]. Nieman et al. [17] reported the detection of early and delayed hypo-enhancement in multidetector CT to be equally as effective as cardiac magnetic resonance imaging (MRI) in determining microvascular obstruction in myocardial infarction. In addition, Akira Sato, MD et al. [18] demonstrated the size of myocardial contrast-delayed enhancement in MDCT scans to significantly predict clinical outcomes in AMI patients. Recently, clinical trials have suggested that coronary computed tomography is an efficient method to detect myocardial infarction and evaluate ACS patients [19,20,21,22]. For better image quality, an ECG-gated, half-rotation reconstruction protocol has been adopted in coronary CT, which may increase the time needed to gather data, limiting CT’s use in the emergency department. Non-ECG-gated CT (NECE-CT) scans have been investigated for ACS patients, but evidence regarding the interpretation of incidental myocardial findings in these scans remains limited. Moreover, some ACS patients present with vague and atypical ACS symptoms that might complicate the interpretation of ACS. In some cases, differential diagnosis of a chest or even abdominal disease with the assistance of non-ECG-gated CT is necessary. With the advancement of CT, the gantry rotation time is 0.33 s in 64-slice MDCT and 0.27 s in 256-slice MDCT model, which enables visualization of the myocardium, and several sporadic reports diagnosing ACS have been published [23,24]. In this study, we aimed to describe the characteristics of myocardial perfusion defects observed on NECE-CT scans and evaluate their concordance with final clinical diagnoses in a retrospective case series of emergency department patients.

## 2. Materials and Methods

### 2.1. Patients

**Figure 1:**
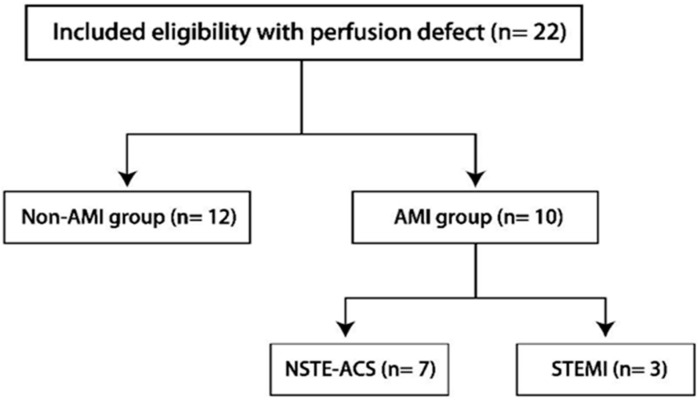
Flowchart of included patients with myocardial perfusion defects in NECE-CT scans.

We retrospectively included all patients between January 2016 and December 2018 who underwent non-ECG-gated contrast-enhanced CT scans in the emergency and radiology departments of Taipei Tzu Chi Hospital that resulted in suspected myocardial perfusion defects noted on their initial NECE-CT reports. These inclusion criteria resulted in a narrow cohort in which to analyze the characteristics of these defects; patients with prior history of AMI or coronary interventions were excluded. The study was approved by the Institutional Review Board of Taipei Tzu Chi Hospital (IRB number: 07-X-119), and informed consent was waived since we only used images and data recorded in medical charts. We retrospectively recruited 22 emergency department patients who underwent NECE-CT scans and showed suspected myocardial infarction in their CT reports. These reports included patients’ symptoms but did not contain any ECG or myocardial enzyme data; therefore, myocardial infarction was suspected due to the myocardial perfusion defects found in either single- or double-phase contrast-enhanced CT. Afterward, we reviewed each patient’s medical chart to collect their clinical parameters, demographic characteristics, cardiac biomarkers, ECG data, and final diagnosis. Only patients presenting with acute chest pain in the emergency department were evaluated using cardiac biomarkers and ECG. The final diagnoses were adjudicated by a retrospective chart review of the attending cardiologist’s discharge diagnosis, based on the 2015 ESC/ACCF/AHA/WHF guidelines, including elevated cardiac cTnI or hs-cTnI during serial follow up, new ST-segment/T wave (ST-T) changes, new left or right bundle branch block (LBBB or RBBB), regional wall motion abnormality in cardiac ultrasound, and intracoronary thrombus determined by angiography or autopsy. The cardiologists were aware of the CT reports when the diagnoses of AMI were made. We retrospectively divided the patients with CT scan cardiac perfusion defects into two groups—an AMI group, with a diagnosis of STEMI or NSTE-ACS, and a control group (Figure 1)—and their demographic data, presenting symptoms, CT images, and scanning protocol were compared.

### 2.2. NECE-CT Imaging Protocol

Owing to the retrospective study design, various CT scanning protocols were applied to these patients. Briefly, non-ECG-gated CE-CT was performed using 16-, 64- (Lightspeed Pro 16, *n* = 1, and Lightspeed VCT, GE Healthcare, Milwaukee, WI, USA, *n* = 20), and 256-slice multidetector CT scanners (Brilliance ICT, Best, The Netherlands, *n* = 1) with the following protocols. Abdomen protocol of Lightspeed Pro 16 (1/22): Tube voltage, 120 kV; tube current, mA modulation technique with a noise index of 11 (mA range: 150–550); gantry rotation time, 0.8 s; pitch, 0.984; collimation, 1.25 mm; reconstruction slice thickness, 5 mm; post-injection delay time (scan), 70 s; window width/ level, 350/45. Abdomen (8/22) and mediastinum (1/22) protocol of Lightspeed VCT: Tube voltage, 120 kV; tube current, mA modulation technique with noise index (NOI) of 11 (mA range: 150–550); gantry rotation time, 0.8 s; pitch, 0.984; collimation width of scanner detector, 1.25 mm; reconstruction slice thickness, 5 mm; post-injection delay time (scan), 70 s and 35 s; window width/ level, 350/45 and 400/40. CTA-chest (11/22) protocol of Lightspeed VCT: Tube voltage, 120 kV; tube current, mA modulation technique with noise index (NOI) of 9.5 (mA range: 220–550); gantry rotation time, 0.5 s; pitch, 0.984; collimation width of scanner detector, 1.25 mm; reconstruction slice thickness, 5 mm. CTA-chest scans used a smart prep technique. The scanning delay was calculated by monitoring the contrast values that increased to about 150 Hounsfield units in the aorta as the regions of interest, and a second scan was performed 30 s after the first scan. The window width/level was 350/45. Abdomen (1/22) protocol of Brilliance iCT: Tube voltage, 120 kV; tube current, mA modulation technique with dose right index (DRI) of 18; gantry rotation time, 0.4 s; pitch, 0.925; collimation width of scanner detector, 0.625 mm; reconstruction slice thickness, 5 mm; post-injection delay time (scan), 75 s and 35 s; window width/ level, 350/60. All of the patients received the contrast medium injection (Omnipaque 350 mg iodine/mL; GE Healthcare, Carrigtohill, Cork, Ireland) intravenously at a rate of 2.0–3.5 mL/s according to their body weight and catheter size, and the total amount of contrast medium administered ranged from 50 mL to 100 mL.

### 2.3. Statistical Analysis

The detailed demographic and laboratory cardiac biomarker data were analyzed by Chi-square analysis and independent-sample *t*-tests via SPSS software (Version 13.0 SPSS Inc., Chicago, IL, USA), while the association between cardiac biomarkers and myocardial perfusion defect size in NECE-CT scans was assessed by linear regression. Statistical significance was defined as a *p*-Value < 0.05. Given the small sample size (*n* = 22), the analysis is primarily descriptive. Inferential statistics (Chi-square, *t*-test) are provided for exploratory purposes only and should be interpreted with caution.

## 3. Results

### 3.1. Patient Characteristics

A total of 22 patients were included in this case series, categorized into AMI and non-AMI groups based on their final diagnoses. As shown in Table 1, the AMI group showed a male predominance and primarily presented with chest pain, followed by syncope. In contrast, abdominal pain was the leading complaint in the non-AMI group.

Regarding initial diagnostic markers, elevated cardiac biomarkers were observed significantly more frequently in the AMI group compared to the non-AMI group, while normal sinus rhythm was the most common ECG finding in both groups. Comorbidities were common, with hypertension being the most prevalent condition across the entire cohort. The detailed clinical characteristics of each patient are presented in Table 2.

### 3.2. Myocardial Infarction in Non-ECG-Gated CT

**Figure 2:**
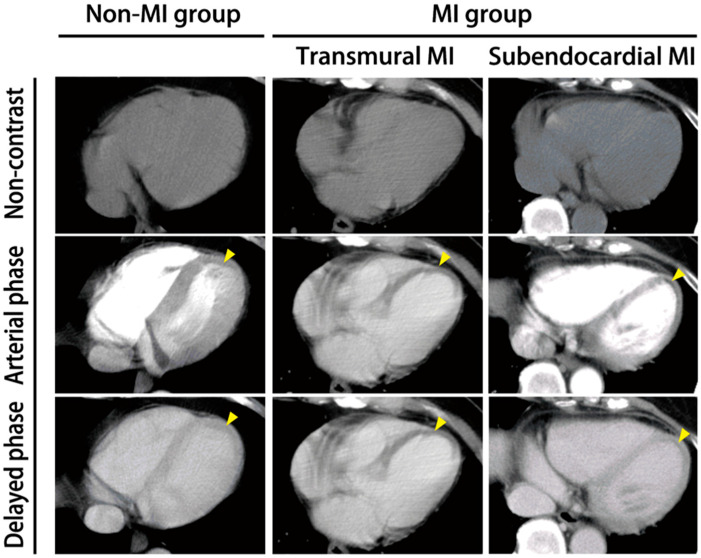
The myocardial perfusion defects in NECE-CT scans of ACS patients. The non-ECG-gated contrast-enhanced computed tomography images reveal whole-layer perfusion defects (arrowhead) in the transmural AMI group and submyocardial perfusion (arrowhead) defects in the subendocardial MI group. In the non-AMI group, myocardial perfusion defects (arrowhead) were noted with hypodensity in the cardiac apex.

Coronary artery calcification was observed more frequently in the AMI group (70%) than the non-AMI group (25%). Specifically, 70% of patients in the AMI group exhibited coronary calcification, compared to only 25% of patients in the non-AMI group. In the non-contrast phase, a low-density myocardial defect usually indicated an old myocardial infarction—in acute myocardial infarction, the hypodensity of the myocardial layer would be detected in the arterial phase and delayed phase. Acute myocardial infarction was divided into two infarct types: transmural and subendocardial (Figure 2). In our cases, subendocardial AMI was noted in eight patients (80%), and transmural in two (20%). The calcification rate of the coronary artery was higher in the AMI group (70%) than in the non-AMI group (25%) (*p* < 0.05), with the left anterior descending artery (LAD) being a major involved coronary artery, followed by the right coronary artery (RCA). The detailed characteristics of the myocardial perfusion defects found in non-ECG-gated CT scans are shown in Table 3.

### 3.3. Right Coronary Artery Occlusion in Non-ECG-Gated CT

**Figure 3:**
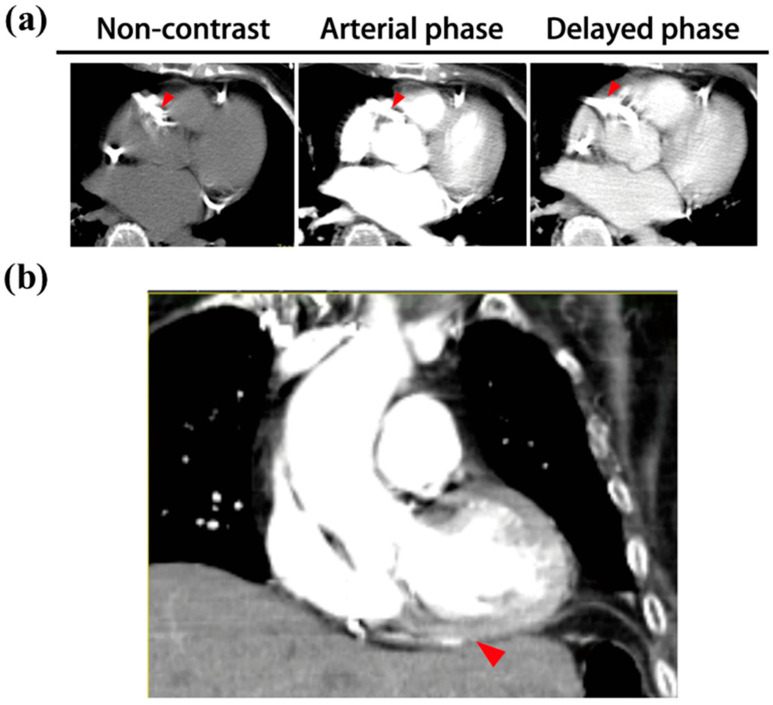
The non-ECG-gated CT scans of a patient with right coronary artery occlusion. (**a**) The right coronary artery in the CT scan showed severe calcification (arrowhead). (**b**) In the coronal plane, the heterogeneous density of the posterior and inferior wall (arrowhead) prevented radiologists evaluating the myocardial infarction area.

In our case analysis, the perfusion defects found via CT scan in cases No. 3 and 8 were not compatible with their PCI findings—there was no significant sign of RCA occlusion in the CT images. The right coronary artery (RCA), which supplies the inferior and posterior wall, is the secondarily involved artery in myocardial infarction. In a non-ECG-gated CT scan, coronary artery calcification is easy to evaluate; however, occlusion of the right coronary artery is difficult to detect. In one case (No. 8) of RCA occlusion, right coronary artery calcification was seen in the non-contrast, arterial, and delayed phases. The myocardial infarction area in the right coronary artery-supplied cardiac walls, the inferior and posterior wall, is difficult to survey due to their heterogeneous density after reconstruction (Figure 3).

## 4. Discussion

Cardiopulmonary diseases, including ACS, pulmonary embolism, and aortic dissection, are life-threatening events in the emergency department which must be rapidly identified and managed. Imaging studies, mainly CT scans, are the gold standard diagnostic tool for their efficacy in assessing pulmonary embolism and aortic dissection. It is challenging to diagnose NSTE-ACS, especially in NSTEMI and the hyper-acute phase, because the coronary arteries are difficult to evaluate via NECE-CT scans. With the rapid progression of CT scanning technology, increasingly high-resolution myocardial images provide more information on vascular perfusion, and CT has been investigated for AMI detection [25,26]. For example, compared with single-photon emission computed tomography (SPECT), which provides a low-resolution image (10 mm × 10 mm × 10 mm), multidetector CT is capable of delivering high-resolution (0.625 mm × 0.625 mm × 0.625 mm) images that can show endocardium details. Therefore, an NECE-CT scan may be helpful in the early detection of major cardiopulmonary diseases.

While functional assessment using FFR-CT has revolutionized non-invasive coronary evaluation, and CT angiography plays a critical role in cerebrovascular ischemia, the utility of standard emergency NECE-CT in myocardial ischemia relies solely on morphological perfusion differences [27]. Understanding these limitations is crucial when comparing NECE-CT to dedicated cardiac imaging.

Although the importance of CT in the diagnosis of AMI has been investigated, showing significant results, most of these studies were based on ECG-gated CT [20,25,26,27,28]. In contrast, we want to address the importance of our research based on NECE-CT. First, it represents actual emergency conditions, in which AMI or even acute chest pain is not diagnosed. Moreover, most of the ECG-gated study protocols include sublingual nitroglycerin medication [25], which might decrease the sensitivity of perfusion defects. In addition, although the ECG-gated CT protocol is optimized for myocardium perfusion detection, it may reduce the sensitivity for detecting pulmonary embolism, aortic dissection, or other diseases that mimic AMI, in which emergent decisions should be made according to the CT. Lastly, two of our AMI patients gave initial impressions of abdominal disease and received CT studies according to abdominal CT protocols. This mixture of various symptoms, signs, and impressions reflects actual situations that emergent radiologists might face.

In this selected cohort, the concordance rate between suspected perfusion defects on NECE-CT and a final diagnosis of AMI was 45% (10/22). However, we found some pearls of wisdom that might improve accurate diagnosis from these CT results. While a higher prevalence of coronary calcification was observed in the MI group, this association does not establish causation but suggests a potential role in enhancing diagnostic confidence. Moreover, although we encountered a heterogeneity of CT protocols due to real emergency practice settings, dual-phase rather than single-phase CT proved to be helpful for the verification of perfusion defects (*p* < 0.05). We want to emphasize the importance of delayed-phase interpretation, in which a slowly perfused myocardium can be excluded from AMI diagnosis. The calcification of coronary arteries also contributes to the precise diagnosis of AMI (*p* < 0.05); although it represents chronic atherosclerotic burden rather than acute occlusion, its presence indicates high cardiovascular risk, and recent studies suggest a link between coronary calcification, plaque vulnerability, and perivascular inflammation [29]. Thus, the presence of calcification on NECE-CT, while not diagnostic of AMI, may serve as a marker of underlying substrate vulnerability. A significant challenge in interpreting perfusion defects is the broad differential diagnosis. Conditions such as stress cardiomyopathy (Takotsubo syndrome) can mimic AMI, with patients presenting with chest pain, ECG changes, and transient perfusion abnormalities [30]. Emergency physicians must remain vigilant to these mimics when interpreting NECE-CT findings.

There are still some important notes for the 55% negative prediction rate. According to clinical findings, in cases where AMI was not the diagnosis, myocardium perfusion insufficiency was still possible. As there is high co-morbidity between CAD and other diseases, further prospective studies with nitroglycerin delivery to verify the chest pain for this group might be necessary.

In a small retrospective study in 2016, Tomomi Watanabe et al. [31] reported that the sensitivity, specificity, positive predictive value, and negative predictive value of NSTE-ACS detection via NECE-CT scans were 84.6%, 90%, 91.7%, and 81.8%, respectively. Our results are similar and support the hypothesis shown by previous studies that NECE-CT scans may serve as an early warning tool before coronary angiography [32]. In four out of ten patients, a myocardial perfusion defect in an NECE-CT scan was the first sign indicating AMI, while initial cardiac enzyme troponin I and ECG still suggested they were MI-negative. This rapid diagnosis of AMI alerts emergency staff to precisely and efficiently initiate coronary angiography, potentially facilitating timely clinical decision-making and reducing diagnostic uncertainty.

Visual myocardial perfusion defects were usually detected in the anterior wall, interventricular septum, or cardiac apex, which the LAD and LCx supply. Paul R. Hilfiker et al. [33] reported a perfusion defect in the posterolateral wall of the left ventricle, and Jean-François Paul showed an anterolateral wall of the left ventricle with a perfusion defect in a CT scan [32]. Myocardial perfusion defects in the LAD and LCx are easy to detect for several reasons: First, the myocardium mass of the left ventricle is more than the right one. Second, the surrounding tissues are complicated and varied, masking the myocardial perfusion defect, and the window of the anterolateral wall or cardiac apex is clear for evaluating the myocardial perfusion. Finally, the direct planes for myocardial perfusion assessment in the posteroinferior wall are the coronal and sagittal planes. However, CT image reconstruction potentially impaired the resolution and increased the presence of artifacts when surveying the myocardial perfusion defect, leading to difficulty diagnosing RCA occlusion via myocardial perfusion defects in a CT image. In our results, right coronary artery (RCA) occlusion was used to detect myocardial infarction in the four cases showing it. However, we retrospectively reviewed the original CT images. It is hard to identify perfusion defects in the right ventricle since the myocardium mass is much lower and motion artifacts are very pronounced. Although an ECG-gated scanning protocol can eliminate these artifacts, the careful trade-off between radiation hazards and potential benefits should be considered.

Previous ECG-gated studies described two myocardial infarction image patterns: transmural and subendocardial [34,35]. Cardiac perfusion is centripetal from the subepicardium to subendocardium in normal physiological conditions. In ischemia, perfusion defects in the subendocardium usually manifest first in CT scans due to the tissue’s lower resistance than the subepicardium [36], meaning that transmural perfusion defects are more significant signs of perfusion abnormalities than subendocardial ones. In our study, the transmural perfusion defects are specific signs in the AMI group (*n* = 2), while subendocardial perfusion defects are found in both groups. After analyzing the subendocardial perfusion defect in the non-AMI group, we found that only four patients received double-phase contrast-enhanced CT. The resulting loss of morphological comparison between the arterial and venous phases may significantly reduce interpretation validity. This finding is compatible with previous ECG-gated studies that describe the delayed phase’s importance [37]. Andreas H. Mahnken et al.’s study [38] found that the pulmonary artery protocol for perfusion defect detection may initiate scans too early to show abnormalities, as the delayed phase plays a critical role in allowing physicians to assess myocardial infarction. In addition, dynamic multi-phase CT imaging provides more valuable information on myocardial perfusion defects than a single arterial phase. Consequently, we strongly suggest that future prospective studies should be conducted with two phases or multi-phase contrast CT to reduce the false-positive rate. Although there is a high false-positive rate in AMI prediction via NECE-CT scans, the value of detecting myocardial perfusion defects is noteworthy. Thrombus formation in the ventricles is one of the critical signs indicating previous AMI [35,36], found two weeks after an acute MI in approximately 10% of AMI patients due to severe local inflammation triggering platelet activation and fibrin deposition. However, we did not observe any delayed enhancement in our study. We propose that this is due to our patient sample coming from the emergency department, which only those with acute AMI attend.

Most importantly, NECE-CT findings should be considered incidental and hypothesis-generating. They cannot and should not replace standard diagnostic modalities such as ECG, troponin assays, or coronary angiography.

This study has several major limitations. First, its sample size is extremely small (*n* = 22), and the retrospective inclusion of only patients with suspected defects introduces significant selection bias. Therefore, diagnostic accuracy metrics (sensitivity/specificity) cannot be calculated for the general population. In addition, our AMI group included a mixture of STEMI and NSTE-ACS patients, which may have introduced clinical and pathophysiological heterogeneity in myocardial perfusion patterns. Due to the limited sample size, we were unable to perform a separate, statistically meaningful analysis for each subgroup. Second, the imaging protocols were highly heterogeneous regarding scanners and timing, reflecting real-world emergency variability but limiting standardization. However, dual-phase rather than single-phase assessment might increase the specificity of AMI diagnosis. Third, image interpretation was not blinded, and no inter-observer variability assessment was performed. Moreover, as a retrospective case series, these results are hypothesis-generating and should not influence current diagnostic algorithms. Future prospective studies with standardized imaging protocols and larger sample sizes are warranted to validate our findings and potentially integrate non-ECG-gated CT scans into diagnostic pathways for AMI [38,39].

## 5. Conclusions

In conclusion, our study suggests that NECE-CT scans may provide incidental supportive findings for AMI diagnosis, especially when combined with assessments of coronary artery calcification. However, due to the limitations of our study and its results being hypothesis-generating, further research via larger, prospective studies is needed before this approach can be recommended in routine clinical practice.

## Figures and Tables

**Table 1:** The clinical characteristics of included patients with perfusion defects in CT scans.

Variables	Total (N = 22)	Non-AMI (N = 12)	AMI (N = 10)
Age, years (mean ± SD)	64.32 ± 14.86	64.00 ± 17.57	64.70 ± 11.73
Gender, *n* (%)			
Male	14 (63.6%)	6 (50.0%)	8 (80.0%)
Female	8 (36.4%)	6 (50.0%)	2 (20.0%)
Chief complaint, *n* (%)			
Chest pain	10 (45.5%)	2 (16.7%)	8 (80.0%)
Abdominal pain	6 (27.3%)	6 (50.0%)	0 (0.00%)
Syncope	2 (9.1%)	0 (0.00%)	2 (20.0%)
Dyspnea	2 (9.1%)	2 (16.7%)	0 (0.00%)
Others	2 (9.1%)	2 (16.7%)	0 (0.00%)
Cardiac biomarkers, *n* (%)			
Elevated	7 (31.8%)	1 (8.3%)	6 (60.0%)
Non-elevated	11 (50.0%)	7 (58.3%)	4 (40.0%)
Electrocardiogram, *n* (%)			
Normal sinus rhythm	12 (54.5%)	5 (41.7%)	7 (70.0%)
ST elevation	2 (9.1%)	0 (0.00%)	2 (20.0%)
Atrial fibrillation	3 (13.6%)	2 (16.7%)	1 (10.0%)
Sinus tachycardia	1 (4.5%)	1 (8.3%)	0 (0.00%)
Diagnosis, *n* (%)			
Cardiovascular disease	11 (50.0%)	1 (8.3%)	10 (100.0%)
STEMI	3 (13.6%)	0 (0.00%)	3 (30.0%)
NSTE-ACS	7 (31.8%)	0 (0.00%)	7 (70.0%)
Gastrointestinal disease	8 (36.4%)	8 (66.7%)	0 (0.00%)
Others	3 (13.6%)	3 (25.0%)	0 (0.00%)
Comorbidity, *n* (%)			
Hypertension	13 (59.1%)	6 (50.0%)	7 (70.0%)
Diabetes mellitus	8 (36.4%)	4 (33.3%)	4 (40.0%)
* Coronary artery, *n* (%)			
Calcification	10 (45.5%)	3 (25.0%)	7 (70.0%)
Non-calcification	12 (54.5%)	9 (75.0%)	3 (30.0%)
* CT phases, *n* (%)			
Single-phase	8 (36.4%)	8 (66.7%)	0 (00.0%)
Double-phase	14 (63.6%)	4 (33.3%)	10 (100.0%)

NSTE-ACS: Non-ST-elevation acute coronary syndrome; STEMI: ST-elevation myocardial infarction; * *p* < 0.05.

**Table 2:** Detailed clinical characteristics of the 22 patients with incidental myocardial perfusion defects.

Patient No.	Age/Sex	Chief Complaint	Initial Troponin 1	Initial ECG	Final Diagnosis
AMI Group					
1	75/M	Syncope	Negative	NSR	NSTE-ACS
2	60/F	Chest pain	Negative	NSR	NSTE-ACS
3	73/F	Chest pain	Positive	NSR	NSTE-ACS
4	50/M	Chest pain	Positive	Afib	NSTE-ACS
5	77/M	Syncope	Positive	NSR	NSTE-ACS
6	56/M	Chest pain	Positive	NSR	NSTE-ACS
7	64/M	Chest pain	Negative	NSR	NSTE-ACS
8	74/M	Chest pain	Positive	ST elevation	STEMI
9	67/M	Chest pain	Positive	ST elevation	STEMI
10	75/M	Chest pain	Negative	NSR	STEMI
Non-AMI Group					
11	62/M	Upper abdominal pain	Negative	NSR	Acute cholecystitis
12	27/M	Gross hematuria	Negative	NSR	Rhabdomyolysis
13	79/M	Chest pain	N/A	Sinus tach	Pancreatic tumor
14	56/M	Chest pain	Negative	N/A	Angina
15	49/F	Lower abdominal pain	N/A	NSR	Colon diverticulitis
16	83/M	Upper abdominal pain	Positive	Afib	Acute cholangitis
17	89/F	Dyspnea	Negative	Afib	Pneumonia
18	71/F	Dyspnea	Negative	N/A	Chronic bronchitis
19	53/M	Difficult swallowing	N/A	N/A	Esophageal cancer
20	70/F	Upper abdominal pain	Negative	NSR	Acute cholecystitis
21	38/F	Upper abdominal pain	N/A	N/A	Acute cholecystitis
22	67/F	Upper abdominal pain	Negative	NSR	Acute gastroenteritis

The AMI group includes patients with a confirmed diagnosis of acute myocardial infarction (NSTE-ACS or STEMI). The non-AMI group includes patients with perfusion defects who were ultimately diagnosed with noncardiac conditions or other cardiac pathologies. Troponin status is classified as “Positive” if levels exceeded the institutional reference range (>0.01 µg/L or equivalent high-sensitivity cutoff) and “Negative” otherwise. Abbreviations: M: male; F: female; NSR: normal sinus rhythm; Afib: atrial fibrillation; Sinus tach: Sinus tachycardia; NSTE-ACS: Non-ST-elevation acute coronary syndrome; STEMI: ST-elevation myocardial infarction; N/A: not available or not performed.

**Table 3:** Characteristics of myocardial perfusion defects in patients with acute myocardial infarction.

Patient	Calcification	Perfusion Defect Location	Infarction Type	PCI Finding
No. 1	LAD, LCX	Lateral wall and apex	Subendocardial AMI	Refuses intervention
No. 2	--	Apex	Subendocardial AMI	LAD spasm and myocardial bridge
No. 3	LAD, LCX, RCA	Anterior wall	Subendocardial AMI	RCA and LAD occlusion
No. 4	LCX	Lateral wall	Subendocardial AMI	LCX occlusion
No. 5	LAD, LCX	Lateral wall, interventricular septum, and apex	Subendocardial AMI	RCA, LCx, and LAD occlusion
No. 6	LAD, LCX	Anterior wall, interventricular septum and apex	Subendocardial AMI	LAD occlusion
No. 7	--	Interventricular septum	Transmural AMI	RCA and LAD occlusion
No. 8	LAD, LCX, RCA	Interventricular septum and apex	Subendocardial AMI	RCA and LAD occlusion
No. 9	--	Anterior wall and interventricular septum	Transmural AMI	LCX occlusion
No. 10	LAD, LCX	Interventricular septum	Subendocardial AMI	LAD occlusion

PCI: percutaneous coronary intervention; RCA: right coronary artery; LCX: left circumflex artery; LAD: left anterior descending artery.

## Data Availability

The datasets used or analyzed during the current study are available from the corresponding author on reasonable request.
